# Supplementary material for: Iterative improvement in the automatic modular design of robot swarms
Source: PeerJ Comput Sci. 2020 Dec 7;6:e322. doi: 10.7717/peerj-cs.322 (PMC7924708; doi:10.7717/peerj-cs.322)
Supplement: Supplemental Information 3 [file peerj-cs-06-322-s003.zip › argos3/doc/api/standalone/a00484_source.html]

ARGoS: plugins/robots/foot-bot/simulator/qtopengl\_footbot.cpp Source File


- Main Page
- Related Pages
- Namespaces
- Classes
- Files

- File List
- File Members

# plugins/robots/foot-bot/simulator/qtopengl\_footbot.cpp

Go to the documentation of this file.

```
00001 
00007 #include "qtopengl_footbot.h"
00008 #include "footbot_entity.h"
00009 #include "footbot_distance_scanner_equipped_entity.h"
00010 #include "footbot_turret_entity.h"
00011 #include <argos3/core/simulator/entity/embodied_entity.h>
00012 #include <argos3/core/utility/math/vector2.h>
00013 #include <argos3/core/utility/math/vector3.h>
00014 #include <argos3/plugins/simulator/entities/led_equipped_entity.h>
00015 #include <argos3/plugins/simulator/entities/gripper_equipped_entity.h>
00016 #include <argos3/plugins/simulator/visualizations/qt-opengl/qtopengl_widget.h>
00017 
00018 namespace argos {
00019 
00020    /****************************************/
00021    /****************************************/
00022 
00023    /* All measures are in meters */
00024 
00025    /* General */
00026    static const Real INTER_MODULE_GAP              = 0.0015f;
00027    static const Real HEIGHT                        = 0.258f;
00028    /* Wheel measures */
00029    static const Real WHEEL_WIDTH                   = 0.022031354f;
00030    static const Real HALF_WHEEL_WIDTH              = WHEEL_WIDTH * 0.5f;
00031    static const Real WHEEL_RADIUS                  = 0.029112741f;
00032    static const Real WHEEL_DIAMETER                = WHEEL_RADIUS * 2.0f;
00033    static const Real INTERWHEEL_DISTANCE           = 0.127f;
00034    static const Real HALF_INTERWHEEL_DISTANCE      = INTERWHEEL_DISTANCE * 0.5f;
00035    /* Track measures */
00036    static const Real INTERTRACK_DISTANCE           = 0.064600514f;
00037    static const Real HALF_INTERTRACK_DISTANCE      = INTERTRACK_DISTANCE * 0.5f;
00038    static const Real TRACK_WHEELS_DISTANCE         = 0.100156677f;
00039    static const Real HALF_TRACK_WHEELS_DISTANCE    = TRACK_WHEELS_DISTANCE * 0.5f;
00040    /* Base module measures */
00041    static const Real BATTERY_SOCKET_ELEVATION      = 0.006f;
00042    static const Real BATTERY_SOCKET_LENGTH         = 0.150302467f;
00043    static const Real HALF_BATTERY_SOCKET_LENGTH    = BATTERY_SOCKET_LENGTH * 0.5f;
00044    static const Real BATTERY_SOCKET_WIDTH          = 0.037600133f;
00045    static const Real HALF_BATTERY_SOCKET_WIDTH     = BATTERY_SOCKET_WIDTH * 0.5f;
00046    static const Real BASE_MODULE_HEIGHT            = 0.0055f;
00047    static const Real BASE_MODULE_RADIUS            = 0.085036758f;
00048    static const Real PROXIMITY_SENSOR_HEIGHT       = 0.004f;  // unused
00049    static const Real PROXIMITY_SENSOR_WIDTH        = 0.007f;  // unused
00050    static const Real PROXIMITY_SENSOR_DEPTH        = 0.0025f; // unused
00051    /* Gripper module measures */
00052    static const Real GRIPPER_MODULE_ELEVATION      = BATTERY_SOCKET_ELEVATION + WHEEL_DIAMETER + BASE_MODULE_HEIGHT + INTER_MODULE_GAP;
00053    static const Real GRIPPER_MODULE_HEIGHT         = 0.027f;
00054    static const Real GRIPPER_MODULE_HALF_HEIGHT    = GRIPPER_MODULE_HEIGHT * 0.5f;
00055    static const Real GRIPPER_MODULE_INNER_RADIUS   = 0.069f;
00056    static const Real GRIPPER_MODULE_BARRIER_HEIGHT = 0.0075f;
00057    static const Real GRIPPER_MECHANICS_LENGTH      = 0.026094485f;
00058    static const Real GRIPPER_MECHANICS_WIDTH       = 0.02f;
00059    static const Real GRIPPER_MECHANICS_HALF_WIDTH  = GRIPPER_MECHANICS_WIDTH * 0.5f;
00060    static const Real GRIPPER_CLAW_LENGTH           = 0.01f;
00061    static const Real GRIPPER_CLAW_WIDTH            = 0.013f;
00062    static const Real GRIPPER_CLAW_HALF_WIDTH       = GRIPPER_CLAW_WIDTH * 0.5f;
00063    static const Real GRIPPER_CLAW_OFFSET           = GRIPPER_MODULE_INNER_RADIUS + GRIPPER_MECHANICS_LENGTH;
00064    static const Real GRIPPER_CLAW_ELEVATION        = GRIPPER_MODULE_ELEVATION + GRIPPER_MODULE_HALF_HEIGHT;
00065    /* RAB measures */
00066    static const Real RAB_ELEVATION                 = GRIPPER_MODULE_ELEVATION + GRIPPER_MODULE_HEIGHT + INTER_MODULE_GAP;
00067    static const Real RAB_HEIGHT                    = 0.0086f;
00068    static const Real RAB_MAX_RADIUS                = 0.08f;
00069    static const Real RAB_MIN_RADIUS                = 0.075f;
00070    /* Distance scanner measures */
00071    static const Real DISTANCE_SCANNER_ELEVATION         = RAB_ELEVATION + RAB_HEIGHT;
00072    static const Real DISTANCE_SCANNER_RADIUS            = 0.05f;
00073    static const Real DISTANCE_SCANNER_HEIGHT            = 0.0235f;
00074    static const Real DISTANCE_SCANNER_SENSOR_WIDTH      = 0.035858477f;
00075    static const Real DISTANCE_SCANNER_SENSOR_HALF_WIDTH = DISTANCE_SCANNER_SENSOR_WIDTH * 0.5f;
00076    static const Real DISTANCE_SCANNER_SENSOR_HEIGHT     = 0.021124933f;
00077    static const Real DISTANCE_SCANNER_SENSOR_DEPTH      = 0.018f;
00078    /* iMX module */
00079    static const Real IMX_MODULE_ELEVATION               = DISTANCE_SCANNER_ELEVATION + DISTANCE_SCANNER_HEIGHT;
00080    static const Real IMX_MODULE_RADIUS                  = 0.065f;
00081    static const Real IMX_MODULE_HEIGHT                  = 0.010199866f;
00082    /* Beacon */
00083    static const Real BEACON_ELEVATION            = IMX_MODULE_ELEVATION + IMX_MODULE_HEIGHT;
00084    static const Real BEACON_RADIUS               = 0.021f;
00085    static const Real BEACON_HEIGHT               = 0.0201f;
00086    /* Camera */
00087    static const Real CAMERA_ELEVATION            = BEACON_ELEVATION + BEACON_HEIGHT;
00088    static const Real CAMERA_RADIUS               = BEACON_RADIUS;
00089    static const Real CAMERA_HEIGHT               = 0.104f;
00090 
00091    /****************************************/
00092    /****************************************/
00093 
00094    CQTOpenGLFootBot::CQTOpenGLFootBot() :
00095       m_unVertices(40),
00096       m_fLEDAngleSlice(360.0f / 12.0f) {
00097       /* Reserve the needed display lists */
00098       m_unLists = glGenLists(13);
00099 
00100       /* Assign indices for better referencing (later) */
00101       m_unBasicWheelList            = m_unLists;
00102       m_unWheelList                 = m_unLists + 1;
00103       m_unTrackList                 = m_unLists + 2;
00104       m_unBaseList                  = m_unLists + 3;
00105       m_unGrippableSliceList        = m_unLists + 4;
00106       m_unGripperMechanicsList      = m_unLists + 5;
00107       m_unGripperClawList           = m_unLists + 6;
00108       m_unRABList                   = m_unLists + 7;
00109       m_unDistanceScannerSensorList = m_unLists + 8;
00110       m_unDistanceScannerList       = m_unLists + 9;
00111       m_unIMXList                   = m_unLists + 10;
00112       m_unBeaconList                = m_unLists + 11;
00113       m_unCameraList                = m_unLists + 12;
00114 
00115       /* Create the materialless wheel display list */
00116       glNewList(m_unBasicWheelList, GL_COMPILE);
00117       MakeWheel();
00118       glEndList();
00119 
00120       /* Create the wheel display list */
00121       glNewList(m_unWheelList, GL_COMPILE);
00122       RenderWheel();
00123       glEndList();
00124 
00125       /* Create the track display list */
00126       glNewList(m_unTrackList, GL_COMPILE);
00127       RenderTrack();
00128       glEndList();
00129 
00130       /* Create the base module display list */
00131       glNewList(m_unBaseList, GL_COMPILE);
00132       RenderBase();
00133       glEndList();
00134 
00135       /* Create the grippable slice display list */
00136       glNewList(m_unGrippableSliceList, GL_COMPILE);
00137       RenderGrippableSlice();
00138       glEndList();
00139 
00140       /* Create the gripper mechanics display list */
00141       glNewList(m_unGripperMechanicsList, GL_COMPILE);
00142       RenderGripperMechanics();
00143       glEndList();
00144 
00145       /* Create the gripper claw display list */
00146       glNewList(m_unGripperClawList, GL_COMPILE);
00147       RenderGripperClaw();
00148       glEndList();
00149 
00150       /* Create the gripper claw display list */
00151       glNewList(m_unRABList, GL_COMPILE);
00152       RenderRAB();
00153       glEndList();
00154 
00155       /* Create the single distance scanner sensor display list */
00156       glNewList(m_unDistanceScannerSensorList, GL_COMPILE);
00157       RenderDistanceScannerSensor();
00158       glEndList();
00159 
00160       /* Create the distance scanner display list */
00161       glNewList(m_unDistanceScannerList, GL_COMPILE);
00162       RenderDistanceScanner();
00163       glEndList();
00164 
00165       /* Create the iMX display list */
00166       glNewList(m_unIMXList, GL_COMPILE);
00167       RenderIMX();
00168       glEndList();
00169 
00170       /* Create the beacon display list */
00171       glNewList(m_unBeaconList, GL_COMPILE);
00172       RenderBeacon();
00173       glEndList();
00174 
00175       /* Create the camera display list */
00176       glNewList(m_unCameraList, GL_COMPILE);
00177       RenderCamera();
00178       glEndList();
00179    }
00180 
00181    /****************************************/
00182    /****************************************/
00183 
00184    CQTOpenGLFootBot::~CQTOpenGLFootBot() {
00185       glDeleteLists(m_unLists, 13);
00186    }
00187 
00188    /****************************************/
00189    /****************************************/
00190 
00191    void CQTOpenGLFootBot::Draw(CFootBotEntity& c_entity) {
00192       /* Place the wheels */
00193       glPushMatrix();
00194       glTranslatef(0.0f, HALF_INTERWHEEL_DISTANCE, 0.0f);
00195       glCallList(m_unWheelList);
00196       glPopMatrix();
00197       glPushMatrix();
00198       glTranslatef(0.0f, -HALF_INTERWHEEL_DISTANCE, 0.0f);
00199       glCallList(m_unWheelList);
00200       glPopMatrix();
00201       /* Place the tracks */
00202       glPushMatrix();
00203       glTranslatef(0.0f, HALF_INTERTRACK_DISTANCE, 0.0f);
00204       glCallList(m_unTrackList);
00205       glPopMatrix();
00206       glPushMatrix();
00207       glTranslatef(0.0f, -HALF_INTERTRACK_DISTANCE, 0.0f);
00208       glCallList(m_unTrackList);
00209       glPopMatrix();
00210       /* Place the tracks */
00211       glCallList(m_unBaseList);
00212       /* Place the gripper module */
00213       glPushMatrix();
00214       /* Read gripper orientation from footbot entity */
00215       GLfloat fGripperOrientation = ToDegrees(c_entity.GetTurretEntity().GetRotation()).GetValue();
00216       glRotatef(fGripperOrientation, 0.0f, 0.0f, 1.0f);
00217       /* Place the grippable part of the gripper module (LEDs) */
00218       glPushMatrix();
00219       CLEDEquippedEntity& cLEDEquippedEntity = c_entity.GetLEDEquippedEntity();
00220       for(UInt32 i = 0; i < 12; i++) {
00221          const CColor& cColor = cLEDEquippedEntity.GetLED(i).GetColor();
00222          glRotatef(m_fLEDAngleSlice, 0.0f, 0.0f, 1.0f);
00223          SetLEDMaterial(cColor.GetRed()   / 255.0f,
00224                         cColor.GetGreen() / 255.0f,
00225                         cColor.GetBlue()  / 255.0f);
00226          glCallList(m_unGrippableSliceList);
00227       }
00228       glPopMatrix();
00229       /* Place the gripper mechanics */
00230       glCallList(m_unGripperMechanicsList);
00231       /* Place the gripper claws */
00232       /* Read the gripper aperture from footbot entity */
00233       GLfloat fGripperAperture = c_entity.GetGripperEquippedEntity().GetLockState() * 90.0f;
00234       glTranslatef(GRIPPER_CLAW_OFFSET, 0.0f, GRIPPER_CLAW_ELEVATION);
00235       glPushMatrix();
00236       glRotatef(fGripperAperture, 0.0f, 1.0f, 0.0f);
00237       glCallList(m_unGripperClawList);
00238       glPopMatrix();
00239       glPushMatrix();
00240       glRotatef(-fGripperAperture, 0.0f, 1.0f, 0.0f);
00241       glCallList(m_unGripperClawList);
00242       glPopMatrix();
00243       glPopMatrix();
00244       /* Place the RAB */
00245       glCallList(m_unRABList);
00246       /* Place the distance scanner */
00247       glCallList(m_unDistanceScannerList);
00248       glPushMatrix();
00249       /* Read dist scanner orientation from footbot entity */
00250       GLfloat fDistanceScannerOrientation = ToDegrees(c_entity.GetDistanceScannerEquippedEntity().GetRotation()).GetValue();
00251       glRotatef(fDistanceScannerOrientation, 0.0f, 0.0f, 1.0f);
00252       glCallList(m_unDistanceScannerSensorList);
00253       glRotatef(90.0f, 0.0f, 0.0f, 1.0f);
00254       glCallList(m_unDistanceScannerSensorList);
00255       glRotatef(90.0f, 0.0f, 0.0f, 1.0f);
00256       glCallList(m_unDistanceScannerSensorList);
00257       glRotatef(90.0f, 0.0f, 0.0f, 1.0f);
00258       glCallList(m_unDistanceScannerSensorList);
00259       glPopMatrix();
00260       /* Place the iMX module */
00261       glCallList(m_unIMXList);
00262       /* Place the beacon */
00263       const CColor& cBeaconColor = cLEDEquippedEntity.GetLED(12).GetColor();
00264       SetLEDMaterial(cBeaconColor.GetRed()   / 255.0f,
00265                      cBeaconColor.GetGreen() / 255.0f,
00266                      cBeaconColor.GetBlue()  / 255.0f);
00267       glCallList(m_unBeaconList);
00268       /* Place the camera */
00269       glCallList(m_unCameraList);
00270    }
00271 
00272    /****************************************/
00273    /****************************************/
00274 
00275    void CQTOpenGLFootBot::MakeWheel() {
00276       /* Right side */
00277       CVector2 cVertex(WHEEL_RADIUS, 0.0f);
00278       CRadians cAngle(CRadians::TWO_PI / m_unVertices);
00279       CVector3 cNormal(-1.0f, -1.0f, 0.0f);
00280       cNormal.Normalize();
00281       glBegin(GL_POLYGON);
00282       for(GLuint i = 0; i <= m_unVertices; i++) {
00283          glNormal3f(cNormal.GetX(), cNormal.GetY(), cNormal.GetZ());
00284          glVertex3f(cVertex.GetX(), -HALF_WHEEL_WIDTH, WHEEL_RADIUS + cVertex.GetY());
00285          cVertex.Rotate(cAngle);
00286          cNormal.RotateY(cAngle);
00287       }
00288       glEnd();
00289       /* Left side */
00290       cVertex.Set(WHEEL_RADIUS, 0.0f);
00291       cNormal.Set(-1.0f, 1.0f, 0.0f);
00292       cNormal.Normalize();
00293       cAngle = -cAngle;
00294       glBegin(GL_POLYGON);
00295       for(GLuint i = 0; i <= m_unVertices; i++) {
00296          glNormal3f(cNormal.GetX(), cNormal.GetY(), cNormal.GetZ());
00297          glVertex3f(cVertex.GetX(), HALF_WHEEL_WIDTH, WHEEL_RADIUS + cVertex.GetY());
00298          cVertex.Rotate(cAngle);
00299          cNormal.RotateY(cAngle);
00300       }
00301       glEnd();
00302       /* Tire */
00303       cNormal.Set(1.0f, 0.0f, 0.0f);
00304       cVertex.Set(WHEEL_RADIUS, 0.0f);
00305       cAngle = -cAngle;
00306       glBegin(GL_QUAD_STRIP);
00307       for(GLuint i = 0; i <= m_unVertices; i++) {
00308          glNormal3f(cNormal.GetX(), cNormal.GetY(), cNormal.GetZ());
00309          glVertex3f(cVertex.GetX(), -HALF_WHEEL_WIDTH, WHEEL_RADIUS + cVertex.GetY());
00310          glVertex3f(cVertex.GetX(),  HALF_WHEEL_WIDTH, WHEEL_RADIUS + cVertex.GetY());
00311          cVertex.Rotate(cAngle);
00312          cNormal.RotateY(cAngle);
00313       }
00314       glEnd();
00315    }
00316 
00317    /****************************************/
00318    /****************************************/
00319 
00320    void CQTOpenGLFootBot::SetWhitePlasticMaterial() {
00321       const GLfloat pfColor[]     = {   1.0f, 1.0f, 1.0f, 1.0f };
00322       const GLfloat pfSpecular[]  = {   0.9f, 0.9f, 0.9f, 1.0f };
00323       const GLfloat pfShininess[] = { 100.0f                   };
00324       const GLfloat pfEmission[]  = {   0.0f, 0.0f, 0.0f, 1.0f };
00325       glMaterialfv(GL_FRONT_AND_BACK, GL_AMBIENT_AND_DIFFUSE, pfColor);
00326       glMaterialfv(GL_FRONT_AND_BACK, GL_SPECULAR,            pfSpecular);
00327       glMaterialfv(GL_FRONT_AND_BACK, GL_SHININESS,           pfShininess);
00328       glMaterialfv(GL_FRONT_AND_BACK, GL_EMISSION,            pfEmission);
00329    }
00330 
00331    /****************************************/
00332    /****************************************/
00333 
00334    void CQTOpenGLFootBot::SetBlackTireMaterial() {
00335       const GLfloat pfColor[]     = { 0.0f, 0.0f, 0.0f, 1.0f };
00336       const GLfloat pfSpecular[]  = { 0.0f, 0.0f, 0.0f, 1.0f };
00337       const GLfloat pfShininess[] = { 0.0f                   };
00338       const GLfloat pfEmission[]  = { 0.0f, 0.0f, 0.0f, 1.0f };
00339       glMaterialfv(GL_FRONT_AND_BACK, GL_AMBIENT_AND_DIFFUSE, pfColor);
00340       glMaterialfv(GL_FRONT_AND_BACK, GL_SPECULAR,            pfSpecular);
00341       glMaterialfv(GL_FRONT_AND_BACK, GL_SHININESS,           pfShininess);
00342       glMaterialfv(GL_FRONT_AND_BACK, GL_EMISSION,            pfEmission);
00343    }
00344 
00345    /****************************************/
00346    /****************************************/
00347 
00348    void CQTOpenGLFootBot::SetCircuitBoardMaterial() {
00349       const GLfloat pfColor[]     = { 0.0f, 0.0f, 1.0f, 1.0f };
00350       const GLfloat pfSpecular[]  = { 0.5f, 0.5f, 1.0f, 1.0f };
00351       const GLfloat pfShininess[] = { 10.0f                  };
00352       const GLfloat pfEmission[]  = { 0.0f, 0.0f, 0.0f, 1.0f };
00353       glMaterialfv(GL_FRONT_AND_BACK, GL_AMBIENT_AND_DIFFUSE, pfColor);
00354       glMaterialfv(GL_FRONT_AND_BACK, GL_SPECULAR,            pfSpecular);
00355       glMaterialfv(GL_FRONT_AND_BACK, GL_SHININESS,           pfShininess);
00356       glMaterialfv(GL_FRONT_AND_BACK, GL_EMISSION,            pfEmission);
00357    }
00358 
00359    /****************************************/
00360    /****************************************/
00361 
00362    void CQTOpenGLFootBot::SetLEDMaterial(GLfloat f_red, GLfloat f_green, GLfloat f_blue) {
00363       const GLfloat fEmissionFactor = 10.0f;
00364       const GLfloat pfColor[]     = {                    f_red,                   f_green,                   f_blue, 1.0f };
00365       const GLfloat pfSpecular[]  = {                     0.0f,                      0.0f,                     0.0f, 1.0f };
00366       const GLfloat pfShininess[] = {                     0.0f                                                            };
00367       const GLfloat pfEmission[]  = {  f_red * fEmissionFactor, f_green * fEmissionFactor, f_blue * fEmissionFactor, 1.0f };
00368       glMaterialfv(GL_FRONT_AND_BACK, GL_AMBIENT_AND_DIFFUSE, pfColor);
00369       glMaterialfv(GL_FRONT_AND_BACK, GL_SPECULAR,            pfSpecular);
00370       glMaterialfv(GL_FRONT_AND_BACK, GL_SHININESS,           pfShininess);
00371       glMaterialfv(GL_FRONT_AND_BACK, GL_EMISSION,            pfEmission);
00372    }
00373 
00374    /****************************************/
00375    /****************************************/
00376 
00377    void CQTOpenGLFootBot::RenderWheel() {
00378       /* Set material */
00379       SetWhitePlasticMaterial();
00380       /* Right side */
00381       CVector2 cVertex(WHEEL_RADIUS, 0.0f);
00382       CRadians cAngle(CRadians::TWO_PI / m_unVertices);
00383       CVector3 cNormal(-1.0f, -1.0f, 0.0f);
00384       cNormal.Normalize();
00385       glBegin(GL_POLYGON);
00386       for(GLuint i = 0; i <= m_unVertices; i++) {
00387          glNormal3f(cNormal.GetX(), cNormal.GetY(), cNormal.GetZ());
00388          glVertex3f(cVertex.GetX(), -HALF_WHEEL_WIDTH, WHEEL_RADIUS + cVertex.GetY());
00389          cVertex.Rotate(cAngle);
00390          cNormal.RotateY(cAngle);
00391       }
00392       glEnd();
00393       /* Left side */
00394       cVertex.Set(WHEEL_RADIUS, 0.0f);
00395       cAngle = -cAngle;
00396       cNormal.Set(-1.0f, 1.0f, 0.0f);
00397       cNormal.Normalize();
00398       glBegin(GL_POLYGON);
00399       for(GLuint i = 0; i <= m_unVertices; i++) {
00400          glNormal3f(cNormal.GetX(), cNormal.GetY(), cNormal.GetZ());
00401          glVertex3f(cVertex.GetX(), HALF_WHEEL_WIDTH, WHEEL_RADIUS + cVertex.GetY());
00402          cVertex.Rotate(cAngle);
00403          cNormal.RotateY(cAngle);
00404       }
00405       glEnd();
00406       /* Tire */
00407       SetBlackTireMaterial();
00408       cNormal.Set(1.0f, 0.0f, 0.0f);
00409       cVertex.Set(WHEEL_RADIUS, 0.0f);
00410       cAngle = -cAngle;
00411       glBegin(GL_QUAD_STRIP);
00412       for(GLuint i = 0; i <= m_unVertices; i++) {
00413          glNormal3f(cNormal.GetX(), cNormal.GetY(), cNormal.GetZ());
00414          glVertex3f(cVertex.GetX(), -HALF_WHEEL_WIDTH, WHEEL_RADIUS + cVertex.GetY());
00415          glVertex3f(cVertex.GetX(),  HALF_WHEEL_WIDTH, WHEEL_RADIUS + cVertex.GetY());
00416          cVertex.Rotate(cAngle);
00417          cNormal.RotateY(cAngle);
00418       }
00419       glEnd();
00420    }
00421 
00422    /****************************************/
00423    /****************************************/
00424 
00425    void CQTOpenGLFootBot::RenderTrack() {
00426       /* Set material */
00427       SetWhitePlasticMaterial();
00428       /* Place two basic wheels */
00429       glPushMatrix();
00430       glTranslatef(HALF_TRACK_WHEELS_DISTANCE, 0.0f, 0.0f);
00431       glCallList(m_unBasicWheelList);
00432       glPopMatrix();
00433       glPushMatrix();
00434       glTranslatef(-HALF_TRACK_WHEELS_DISTANCE, 0.0f, 0.0f);
00435       glCallList(m_unBasicWheelList);
00436       glPopMatrix();
00437       /* Render the track itself */
00438       /* Set material */
00439       SetBlackTireMaterial();
00440       glEnable(GL_POLYGON_OFFSET_FILL); // Correct flickering due to overlapping surfaces
00441       glPolygonOffset(-0.1, 1.0);      // Correct flickering due to overlapping surfaces
00442       /* Top part */
00443       glBegin(GL_POLYGON);
00444       glVertex3f(-HALF_TRACK_WHEELS_DISTANCE, -HALF_WHEEL_WIDTH, WHEEL_DIAMETER);
00445       glVertex3f( HALF_TRACK_WHEELS_DISTANCE, -HALF_WHEEL_WIDTH, WHEEL_DIAMETER);
00446       glVertex3f( HALF_TRACK_WHEELS_DISTANCE,  HALF_WHEEL_WIDTH, WHEEL_DIAMETER);
00447       glVertex3f(-HALF_TRACK_WHEELS_DISTANCE,  HALF_WHEEL_WIDTH, WHEEL_DIAMETER);
00448       glEnd();
00449       /* Bottom part */
00450       glBegin(GL_POLYGON);
00451       glVertex3f(-HALF_TRACK_WHEELS_DISTANCE, -HALF_WHEEL_WIDTH, 0.0f);
00452       glVertex3f( HALF_TRACK_WHEELS_DISTANCE, -HALF_WHEEL_WIDTH, 0.0f);
00453       glVertex3f( HALF_TRACK_WHEELS_DISTANCE,  HALF_WHEEL_WIDTH, 0.0f);
00454       glVertex3f(-HALF_TRACK_WHEELS_DISTANCE,  HALF_WHEEL_WIDTH, 0.0f);
00455       glEnd();
00456       /* Round parts */
00457       CVector2 cVertex(0.0f, WHEEL_RADIUS);
00458       CVector2 cNormal(0.0f, 1.0f);
00459       CRadians cAngle(CRadians::TWO_PI / m_unVertices);
00460       /* Front */
00461       glBegin(GL_QUAD_STRIP);
00462       for(GLuint i = 0; i <= m_unVertices / 2; i++) {
00463          glNormal3f(cNormal.GetX(), 0.0f, cNormal.GetY());
00464          glVertex3f(-HALF_TRACK_WHEELS_DISTANCE + cVertex.GetX(), -HALF_WHEEL_WIDTH, WHEEL_RADIUS + cVertex.GetY());
00465          glVertex3f(-HALF_TRACK_WHEELS_DISTANCE + cVertex.GetX(),  HALF_WHEEL_WIDTH, WHEEL_RADIUS + cVertex.GetY());
00466          cVertex.Rotate(cAngle);
00467          cNormal.Rotate(cAngle);
00468       }
00469       glEnd();
00470       /* Back */
00471       glBegin(GL_QUAD_STRIP);
00472       for(GLuint i = 0; i <= m_unVertices / 2; i++) {
00473          glNormal3f(cNormal.GetX(), 0.0f, cNormal.GetY());
00474          glVertex3f(HALF_TRACK_WHEELS_DISTANCE + cVertex.GetX(), -HALF_WHEEL_WIDTH, WHEEL_RADIUS + cVertex.GetY());
00475          glVertex3f(HALF_TRACK_WHEELS_DISTANCE + cVertex.GetX(),  HALF_WHEEL_WIDTH, WHEEL_RADIUS + cVertex.GetY());
00476          cVertex.Rotate(cAngle);
00477          cNormal.Rotate(cAngle);
00478       }
00479       glEnd();
00480       glDisable(GL_POLYGON_OFFSET_FILL); // Not needed anymore
00481    }
00482 
00483    /****************************************/
00484    /****************************************/
00485 
00486    void CQTOpenGLFootBot::RenderBase() {
00487       /* Battery socket */
00488       /* Set material */
00489       SetWhitePlasticMaterial();
00490       /* This part covers the top and bottom faces (parallel to XY) */
00491       glBegin(GL_QUADS);
00492       /* Bottom face */
00493       glNormal3f(0.0f, 0.0f, -1.0f);
00494       glVertex3f( HALF_BATTERY_SOCKET_LENGTH,  HALF_BATTERY_SOCKET_WIDTH, BATTERY_SOCKET_ELEVATION);
00495       glVertex3f( HALF_BATTERY_SOCKET_LENGTH, -HALF_BATTERY_SOCKET_WIDTH, BATTERY_SOCKET_ELEVATION);
00496       glVertex3f(-HALF_BATTERY_SOCKET_LENGTH, -HALF_BATTERY_SOCKET_WIDTH, BATTERY_SOCKET_ELEVATION);
00497       glVertex3f(-HALF_BATTERY_SOCKET_LENGTH,  HALF_BATTERY_SOCKET_WIDTH, BATTERY_SOCKET_ELEVATION);
00498       /* Top face */
00499       glNormal3f(0.0f, 0.0f, 1.0f);
00500       glVertex3f(-HALF_BATTERY_SOCKET_LENGTH, -HALF_BATTERY_SOCKET_WIDTH, BATTERY_SOCKET_ELEVATION + WHEEL_DIAMETER);
00501       glVertex3f( HALF_BATTERY_SOCKET_LENGTH, -HALF_BATTERY_SOCKET_WIDTH, BATTERY_SOCKET_ELEVATION + WHEEL_DIAMETER);
00502       glVertex3f( HALF_BATTERY_SOCKET_LENGTH,  HALF_BATTERY_SOCKET_WIDTH, BATTERY_SOCKET_ELEVATION + WHEEL_DIAMETER);
00503       glVertex3f(-HALF_BATTERY_SOCKET_LENGTH,  HALF_BATTERY_SOCKET_WIDTH, BATTERY_SOCKET_ELEVATION + WHEEL_DIAMETER);
00504       glEnd();
00505       /* This part covers the faces (South, East, North, West) */
00506       glBegin(GL_QUAD_STRIP);
00507       /* Starting side */
00508       glNormal3f(-1.0f, 0.0f, 0.0f);
00509       glVertex3f(-HALF_BATTERY_SOCKET_LENGTH, -HALF_BATTERY_SOCKET_WIDTH, BATTERY_SOCKET_ELEVATION + WHEEL_DIAMETER);
00510       glVertex3f(-HALF_BATTERY_SOCKET_LENGTH, -HALF_BATTERY_SOCKET_WIDTH, BATTERY_SOCKET_ELEVATION);
00511       /* South face */
00512       glVertex3f( HALF_BATTERY_SOCKET_LENGTH, -HALF_BATTERY_SOCKET_WIDTH, BATTERY_SOCKET_ELEVATION + WHEEL_DIAMETER);
00513       glVertex3f( HALF_BATTERY_SOCKET_LENGTH, -HALF_BATTERY_SOCKET_WIDTH, BATTERY_SOCKET_ELEVATION);
00514       /* East face */
00515       glNormal3f(0.0f, -1.0f, 0.0f);
00516       glVertex3f( HALF_BATTERY_SOCKET_LENGTH,  HALF_BATTERY_SOCKET_WIDTH, BATTERY_SOCKET_ELEVATION + WHEEL_DIAMETER);
00517       glVertex3f( HALF_BATTERY_SOCKET_LENGTH,  HALF_BATTERY_SOCKET_WIDTH, BATTERY_SOCKET_ELEVATION);
00518       /* North face */
00519       glNormal3f(1.0f, 0.0f, 0.0f);
00520       glVertex3f(-HALF_BATTERY_SOCKET_LENGTH,  HALF_BATTERY_SOCKET_WIDTH, BATTERY_SOCKET_ELEVATION + WHEEL_DIAMETER);
00521       glVertex3f(-HALF_BATTERY_SOCKET_LENGTH,  HALF_BATTERY_SOCKET_WIDTH, BATTERY_SOCKET_ELEVATION);
00522       /* West face */
00523       glNormal3f(0.0f, 1.0f, 0.0f);
00524       glVertex3f(-HALF_BATTERY_SOCKET_LENGTH, -HALF_BATTERY_SOCKET_WIDTH, BATTERY_SOCKET_ELEVATION + WHEEL_DIAMETER);
00525       glVertex3f(-HALF_BATTERY_SOCKET_LENGTH, -HALF_BATTERY_SOCKET_WIDTH, BATTERY_SOCKET_ELEVATION);
00526       glEnd();
00527       /* Circuit board */
00528       CVector2 cVertex(BASE_MODULE_RADIUS, 0.0f);
00529       CRadians cAngle(-CRadians::TWO_PI / m_unVertices);
00530       /* Bottom part */
00531       glBegin(GL_POLYGON);
00532       glNormal3f(0.0f, 0.0f, -1.0f);
00533       for(GLuint i = 0; i <= m_unVertices; i++) {
00534          glVertex3f(cVertex.GetX(), cVertex.GetY(), BATTERY_SOCKET_ELEVATION + WHEEL_DIAMETER);
00535          cVertex.Rotate(cAngle);
00536       }
00537       glEnd();
00538       /* Side surface */
00539       cAngle = -cAngle;
00540       CVector2 cNormal(1.0f, 0.0f);
00541       cVertex.Set(BASE_MODULE_RADIUS, 0.0f);
00542       glBegin(GL_QUAD_STRIP);
00543       for(GLuint i = 0; i <= m_unVertices; i++) {
00544          glNormal3f(cNormal.GetX(), cNormal.GetY(), 0.0f);
00545          glVertex3f(cVertex.GetX(), cVertex.GetY(), BATTERY_SOCKET_ELEVATION + WHEEL_DIAMETER + BASE_MODULE_HEIGHT);
00546          glVertex3f(cVertex.GetX(), cVertex.GetY(), BATTERY_SOCKET_ELEVATION + WHEEL_DIAMETER);
00547          cVertex.Rotate(cAngle);
00548          cNormal.Rotate(cAngle);
00549       }
00550       glEnd();
00551       /* Top part */
00552       /* Set material */
00553       SetCircuitBoardMaterial();
00554       glBegin(GL_POLYGON);
00555       glNormal3f(0.0f, 0.0f, 1.0f);
00556       cVertex.Set(BASE_MODULE_RADIUS, 0.0f);
00557       for(GLuint i = 0; i <= m_unVertices; i++) {
00558          glVertex3f(cVertex.GetX(), cVertex.GetY(), BATTERY_SOCKET_ELEVATION + WHEEL_DIAMETER + BASE_MODULE_HEIGHT);
00559          cVertex.Rotate(cAngle);
00560       }
00561       glEnd();
00562    }
00563 
00564    /****************************************/
00565    /****************************************/
00566 
00567    void CQTOpenGLFootBot::RenderGrippableSlice() {
00568       glDisable(GL_CULL_FACE); // This way we can see both faces of the grippable slice
00569       /* Bottom part */
00570       CVector2 cVertex(BASE_MODULE_RADIUS, 0.0f);
00571       cVertex.Rotate(-CRadians::TWO_PI / 12);
00572       CRadians cAngle(CRadians::TWO_PI / (m_unVertices * 12));
00573       glBegin(GL_TRIANGLE_FAN);
00574       glNormal3f(0.0f, 0.0f, -1.0f);
00575       glVertex3f(0.0f, 0.0f, GRIPPER_MODULE_ELEVATION);
00576       for(GLuint i = 0; i <= m_unVertices; i++) {
00577          glVertex3f(cVertex.GetX(), cVertex.GetY(), GRIPPER_MODULE_ELEVATION);
00578          cVertex.Rotate(cAngle);
00579       }
00580       glEnd();
00581       cAngle = -CRadians::TWO_PI / (m_unVertices * 12);
00582       /* Side barrier surface (bottom) */
00583       CVector2 cNormal(1.0f, 0.0f);
00584       cVertex.Set(BASE_MODULE_RADIUS, 0.0f);
00585       glBegin(GL_QUAD_STRIP);
00586       for(GLuint i = 0; i <= m_unVertices; i++) {
00587          glNormal3f(cVertex.GetX(), cVertex.GetY(), 0.0f);
00588          glVertex3f(cVertex.GetX(), cVertex.GetY(), GRIPPER_MODULE_ELEVATION + GRIPPER_MODULE_BARRIER_HEIGHT);
00589          glVertex3f(cVertex.GetX(), cVertex.GetY(), GRIPPER_MODULE_ELEVATION);
00590          cVertex.Rotate(cAngle);
00591          cNormal.Rotate(cAngle);
00592       }
00593       glEnd();
00594       /* Side barrier surface (top) */
00595       cNormal.Set(1.0f, 0.0f);
00596       cVertex.Set(BASE_MODULE_RADIUS, 0.0f);
00597       glBegin(GL_QUAD_STRIP);
00598       for(GLuint i = 0; i <= m_unVertices; i++) {
00599          glNormal3f(cVertex.GetX(), cVertex.GetY(), 0.0f);
00600          glVertex3f(cVertex.GetX(), cVertex.GetY(), GRIPPER_MODULE_ELEVATION + GRIPPER_MODULE_HEIGHT);
00601          glVertex3f(cVertex.GetX(), cVertex.GetY(), GRIPPER_MODULE_ELEVATION + GRIPPER_MODULE_HEIGHT - GRIPPER_MODULE_BARRIER_HEIGHT);
00602          cVertex.Rotate(cAngle);
00603          cNormal.Rotate(cAngle);
00604       }
00605       glEnd();
00606       /* Internal rod (bottom) */
00607       cNormal.Set(BASE_MODULE_RADIUS - GRIPPER_MODULE_INNER_RADIUS,
00608                   GRIPPER_MODULE_HALF_HEIGHT);
00609       cNormal.Normalize();
00610       cVertex.Set(BASE_MODULE_RADIUS, 0.0f);
00611       CVector2 cVertex2(GRIPPER_MODULE_INNER_RADIUS, 0.0f);
00612       glBegin(GL_QUAD_STRIP);
00613       for(GLuint i = 0; i <= m_unVertices; i++) {
00614          glNormal3f(cVertex.GetX(), cVertex.GetY(), 0.0f);
00615          glVertex3f(cVertex2.GetX(), cVertex2.GetY(), GRIPPER_MODULE_ELEVATION + GRIPPER_MODULE_HALF_HEIGHT);
00616          glVertex3f(cVertex.GetX(), cVertex.GetY(), GRIPPER_MODULE_ELEVATION);
00617          cVertex.Rotate(cAngle);
00618          cVertex2.Rotate(cAngle);
00619          cNormal.Rotate(cAngle);
00620       }
00621       glEnd();
00622       /* Internal rod (top) */
00623       cNormal.Set( BASE_MODULE_RADIUS - GRIPPER_MODULE_INNER_RADIUS,
00624                   -GRIPPER_MODULE_HALF_HEIGHT);
00625       cNormal.Normalize();
00626       cVertex.Set(BASE_MODULE_RADIUS, 0.0f);
00627       cVertex2.Set(GRIPPER_MODULE_INNER_RADIUS, 0.0f);
00628       glBegin(GL_QUAD_STRIP);
00629       for(GLuint i = 0; i <= m_unVertices; i++) {
00630          glNormal3f(cVertex.GetX(), cVertex.GetY(), 0.0f);
00631          glVertex3f(cVertex.GetX(), cVertex.GetY(), GRIPPER_MODULE_ELEVATION + GRIPPER_MODULE_HEIGHT);
00632          glVertex3f(cVertex2.GetX(), cVertex2.GetY(), GRIPPER_MODULE_ELEVATION + GRIPPER_MODULE_HALF_HEIGHT);
00633          cVertex.Rotate(cAngle);
00634          cVertex2.Rotate(cAngle);
00635          cNormal.Rotate(cAngle);
00636       }
00637       glEnd();
00638       /* Top part */
00639       cVertex.Set(BASE_MODULE_RADIUS, 0.0f);
00640       glBegin(GL_TRIANGLE_FAN);
00641       glNormal3f(0.0f, 0.0f, 1.0f);
00642       glVertex3f(0.0f, 0.0f, GRIPPER_MODULE_ELEVATION + GRIPPER_MODULE_HEIGHT);
00643       for(GLuint i = 0; i <= m_unVertices; i++) {
00644          glVertex3f(cVertex.GetX(), cVertex.GetY(), GRIPPER_MODULE_ELEVATION + GRIPPER_MODULE_HEIGHT);
00645          cVertex.Rotate(cAngle);
00646       }
00647       glEnd();
00648       glEnable(GL_CULL_FACE); // Back to normal setting: cull back faces
00649    }
00650 
00651    /****************************************/
00652    /****************************************/
00653 
00654    void CQTOpenGLFootBot::RenderGripperMechanics() {
00655       /* Set material */
00656       const GLfloat pfColor[]     = { 0.0f, 0.0f, 0.0f, 1.0f };
00657       const GLfloat pfSpecular[]  = { 0.0f, 0.0f, 0.0f, 1.0f };
00658       const GLfloat pfShininess[] = { 0.0f                   };
00659       const GLfloat pfEmission[]  = { 0.0f, 0.0f, 0.0f, 1.0f };
00660       glMaterialfv(GL_FRONT_AND_BACK, GL_AMBIENT_AND_DIFFUSE, pfColor);
00661       glMaterialfv(GL_FRONT_AND_BACK, GL_SPECULAR,            pfSpecular);
00662       glMaterialfv(GL_FRONT_AND_BACK, GL_SHININESS,           pfShininess);
00663       glMaterialfv(GL_FRONT_AND_BACK, GL_EMISSION,            pfEmission);
00664       /* This part covers the top and bottom faces (parallel to XY) */
00665       glEnable(GL_POLYGON_OFFSET_FILL); // Correct flickering due to overlapping surfaces
00666       glPolygonOffset(-0.1, 1.0);      // Correct flickering due to overlapping surfaces
00667       glBegin(GL_QUADS);
00668       /* Bottom face */
00669       glNormal3f(0.0f, 0.0f, -1.0f);
00670       glVertex3f(GRIPPER_MECHANICS_LENGTH + GRIPPER_MODULE_INNER_RADIUS,  GRIPPER_MECHANICS_HALF_WIDTH, GRIPPER_MODULE_ELEVATION);
00671       glVertex3f(GRIPPER_MECHANICS_LENGTH + GRIPPER_MODULE_INNER_RADIUS, -GRIPPER_MECHANICS_HALF_WIDTH, GRIPPER_MODULE_ELEVATION);
00672       glVertex3f(                           GRIPPER_MODULE_INNER_RADIUS, -GRIPPER_MECHANICS_HALF_WIDTH, GRIPPER_MODULE_ELEVATION);
00673       glVertex3f(                           GRIPPER_MODULE_INNER_RADIUS,  GRIPPER_MECHANICS_HALF_WIDTH, GRIPPER_MODULE_ELEVATION);
00674       /* Top face */
00675       glNormal3f(0.0f, 0.0f, 1.0f);
00676       glVertex3f(                           GRIPPER_MODULE_INNER_RADIUS, -GRIPPER_MECHANICS_HALF_WIDTH, GRIPPER_MODULE_ELEVATION + GRIPPER_MODULE_HEIGHT);
00677       glVertex3f(GRIPPER_MECHANICS_LENGTH + GRIPPER_MODULE_INNER_RADIUS, -GRIPPER_MECHANICS_HALF_WIDTH, GRIPPER_MODULE_ELEVATION + GRIPPER_MODULE_HEIGHT);
00678       glVertex3f(GRIPPER_MECHANICS_LENGTH + GRIPPER_MODULE_INNER_RADIUS,  GRIPPER_MECHANICS_HALF_WIDTH, GRIPPER_MODULE_ELEVATION + GRIPPER_MODULE_HEIGHT);
00679       glVertex3f(                           GRIPPER_MODULE_INNER_RADIUS,  GRIPPER_MECHANICS_HALF_WIDTH, GRIPPER_MODULE_ELEVATION + GRIPPER_MODULE_HEIGHT);
00680       glEnd();
00681       glDisable(GL_POLYGON_OFFSET_FILL); // Not needed anymore
00682       /* This part covers the faces (South, East, North, West) */
00683       glBegin(GL_QUAD_STRIP);
00684       glNormal3f(0.0f, -1.0f, 0.0f);
00685       /* Starting vertex */
00686       glVertex3f(                           GRIPPER_MODULE_INNER_RADIUS, -GRIPPER_MECHANICS_HALF_WIDTH, GRIPPER_MODULE_ELEVATION + GRIPPER_MODULE_HEIGHT);
00687       glVertex3f(                           GRIPPER_MODULE_INNER_RADIUS, -GRIPPER_MECHANICS_HALF_WIDTH, GRIPPER_MODULE_ELEVATION);
00688       /* East face */
00689       glNormal3f(1.0f, 0.0f, 0.0f);
00690       glVertex3f(GRIPPER_MECHANICS_LENGTH + GRIPPER_MODULE_INNER_RADIUS, -GRIPPER_MECHANICS_HALF_WIDTH, GRIPPER_MODULE_ELEVATION + GRIPPER_MODULE_HEIGHT);
00691       glVertex3f(GRIPPER_MECHANICS_LENGTH + GRIPPER_MODULE_INNER_RADIUS, -GRIPPER_MECHANICS_HALF_WIDTH, GRIPPER_MODULE_ELEVATION);
00692       /* North face */
00693       glNormal3f(0.0f, 1.0f, 0.0f);
00694       glVertex3f(GRIPPER_MECHANICS_LENGTH + GRIPPER_MODULE_INNER_RADIUS,  GRIPPER_MECHANICS_HALF_WIDTH, GRIPPER_MODULE_ELEVATION + GRIPPER_MODULE_HEIGHT);
00695       glVertex3f(GRIPPER_MECHANICS_LENGTH + GRIPPER_MODULE_INNER_RADIUS,  GRIPPER_MECHANICS_HALF_WIDTH, GRIPPER_MODULE_ELEVATION);
00696       /* West face */
00697       glNormal3f(-1.0f, 0.0f, 0.0f);
00698       glVertex3f(                           GRIPPER_MODULE_INNER_RADIUS,  GRIPPER_MECHANICS_HALF_WIDTH, GRIPPER_MODULE_ELEVATION + GRIPPER_MODULE_HEIGHT);
00699       glVertex3f(                           GRIPPER_MODULE_INNER_RADIUS,  GRIPPER_MECHANICS_HALF_WIDTH, GRIPPER_MODULE_ELEVATION);
00700       glEnd();
00701    }
00702 
00703    /****************************************/
00704    /****************************************/
00705 
00706    void CQTOpenGLFootBot::RenderGripperClaw() {
00707       /* Set material */
00708       const GLfloat pfColor[]     = { 0.5f, 0.5f, 0.5f, 1.0f };
00709       const GLfloat pfSpecular[]  = { 0.5f, 0.5f, 0.5f, 1.0f };
00710       const GLfloat pfShininess[] = { 10.0f                  };
00711       const GLfloat pfEmission[]  = { 0.0f, 0.0f, 0.0f, 1.0f };
00712       glMaterialfv(GL_FRONT_AND_BACK, GL_AMBIENT_AND_DIFFUSE, pfColor);
00713       glMaterialfv(GL_FRONT_AND_BACK, GL_SPECULAR,            pfSpecular);
00714       glMaterialfv(GL_FRONT_AND_BACK, GL_SHININESS,           pfShininess);
00715       glMaterialfv(GL_FRONT_AND_BACK, GL_EMISSION,            pfEmission);
00716       /* Draw the claw */
00717       glDisable(GL_CULL_FACE); // In this way, we can see both sides of the claw
00718       glNormal3f(0.0f, 0.0f, 1.0f);
00719       glBegin(GL_QUADS);
00720       glVertex3f(               0.0f,  GRIPPER_CLAW_HALF_WIDTH, 0.0f);
00721       glVertex3f(               0.0f, -GRIPPER_CLAW_HALF_WIDTH, 0.0f);
00722       glVertex3f(GRIPPER_CLAW_LENGTH, -GRIPPER_CLAW_HALF_WIDTH, 0.0f);
00723       glVertex3f(GRIPPER_CLAW_LENGTH,  GRIPPER_CLAW_HALF_WIDTH, 0.0f);
00724       glEnd();
00725       glEnable(GL_CULL_FACE); // Restore back face culling
00726    }
00727 
00728    /****************************************/
00729    /****************************************/
00730 
00731    void CQTOpenGLFootBot::RenderRAB() {
00732       /* Set material */
00733       SetWhitePlasticMaterial();
00734       /* Bottom part */
00735       CVector2 cVertex(RAB_MAX_RADIUS, 0.0f);
00736       CRadians cAngle(-CRadians::TWO_PI / m_unVertices);
00737       glBegin(GL_POLYGON);
00738       glNormal3f(0.0f, 0.0f, -1.0f);
00739       for(GLuint i = 0; i <= m_unVertices; i++) {
00740          glVertex3f(cVertex.GetX(), cVertex.GetY(), RAB_ELEVATION);
00741          cVertex.Rotate(cAngle);
00742       }
00743       glEnd();
00744       /* Side surface */
00745       cAngle = -cAngle;
00746       CVector3 cNormal(RAB_MAX_RADIUS - RAB_MIN_RADIUS, 0.0f, RAB_HEIGHT);
00747       cNormal.Normalize();
00748       cVertex.Set(RAB_MAX_RADIUS, 0.0f);
00749       CVector2 cVertex2(RAB_MIN_RADIUS, 0.0f);
00750       glBegin(GL_QUAD_STRIP);
00751       for(GLuint i = 0; i <= m_unVertices; i++) {
00752          glNormal3f(cNormal.GetX(), cNormal.GetY(), cNormal.GetZ());
00753          glVertex3f(cVertex2.GetX(), cVertex2.GetY(), RAB_ELEVATION + RAB_HEIGHT);
00754          glVertex3f(cVertex.GetX(), cVertex.GetY(), RAB_ELEVATION);
00755          cVertex.Rotate(cAngle);
00756          cVertex2.Rotate(cAngle);
00757          cNormal.RotateZ(cAngle);
00758       }
00759       glEnd();
00760       /* Top part */
00761       /* Set material */
00762       SetCircuitBoardMaterial();
00763       cVertex.Set(RAB_MIN_RADIUS, 0.0f);
00764       glBegin(GL_POLYGON);
00765       glNormal3f(0.0f, 0.0f, 1.0f);
00766       for(GLuint i = 0; i <= m_unVertices; i++) {
00767          glVertex3f(cVertex.GetX(), cVertex.GetY(), RAB_ELEVATION + RAB_HEIGHT);
00768          cVertex.Rotate(cAngle);
00769       }
00770       glEnd();
00771    }
00772 
00773    /****************************************/
00774    /****************************************/
00775 
00776    void CQTOpenGLFootBot::RenderDistanceScannerSensor() {
00777       /* Set material */
00778       const GLfloat pfColor[]     = { 0.0f, 0.0f, 0.0f, 1.0f };
00779       const GLfloat pfSpecular[]  = { 0.0f, 0.0f, 0.0f, 1.0f };
00780       const GLfloat pfShininess[] = { 0.0f                   };
00781       const GLfloat pfEmission[]  = { 0.0f, 0.0f, 0.0f, 1.0f };
00782       glMaterialfv(GL_FRONT_AND_BACK, GL_AMBIENT_AND_DIFFUSE, pfColor);
00783       glMaterialfv(GL_FRONT_AND_BACK, GL_SPECULAR,            pfSpecular);
00784       glMaterialfv(GL_FRONT_AND_BACK, GL_SHININESS,           pfShininess);
00785       glMaterialfv(GL_FRONT_AND_BACK, GL_EMISSION,            pfEmission);
00786       /* This part covers the top and bottom faces (parallel to XY) */
00787       glBegin(GL_QUADS);
00788       /* Bottom face */
00789       glNormal3f(0.0f, 0.0f, -1.0f);
00790       glVertex3f(DISTANCE_SCANNER_SENSOR_DEPTH + DISTANCE_SCANNER_RADIUS,  DISTANCE_SCANNER_SENSOR_HALF_WIDTH, DISTANCE_SCANNER_ELEVATION);
00791       glVertex3f(DISTANCE_SCANNER_SENSOR_DEPTH + DISTANCE_SCANNER_RADIUS, -DISTANCE_SCANNER_SENSOR_HALF_WIDTH, DISTANCE_SCANNER_ELEVATION);
00792       glVertex3f(                                DISTANCE_SCANNER_RADIUS, -DISTANCE_SCANNER_SENSOR_HALF_WIDTH, DISTANCE_SCANNER_ELEVATION);
00793       glVertex3f(                                DISTANCE_SCANNER_RADIUS,  DISTANCE_SCANNER_SENSOR_HALF_WIDTH, DISTANCE_SCANNER_ELEVATION);
00794       /* Top face */
00795       glNormal3f(0.0f, 0.0f, 1.0f);
00796       glVertex3f(                                DISTANCE_SCANNER_RADIUS, -DISTANCE_SCANNER_SENSOR_HALF_WIDTH, DISTANCE_SCANNER_ELEVATION + DISTANCE_SCANNER_HEIGHT);
00797       glVertex3f(DISTANCE_SCANNER_SENSOR_DEPTH + DISTANCE_SCANNER_RADIUS, -DISTANCE_SCANNER_SENSOR_HALF_WIDTH, DISTANCE_SCANNER_ELEVATION + DISTANCE_SCANNER_HEIGHT);
00798       glVertex3f(DISTANCE_SCANNER_SENSOR_DEPTH + DISTANCE_SCANNER_RADIUS,  DISTANCE_SCANNER_SENSOR_HALF_WIDTH, DISTANCE_SCANNER_ELEVATION + DISTANCE_SCANNER_HEIGHT);
00799       glVertex3f(                                DISTANCE_SCANNER_RADIUS,  DISTANCE_SCANNER_SENSOR_HALF_WIDTH, DISTANCE_SCANNER_ELEVATION + DISTANCE_SCANNER_HEIGHT);
00800       glEnd();
00801       glDisable(GL_POLYGON_OFFSET_FILL); // Not needed anymore
00802       /* This part covers the faces (South, East, North, West) */
00803       glBegin(GL_QUAD_STRIP);
00804       glNormal3f(0.0f, -1.0f, 0.0f);
00805       /* Starting vertex */
00806       glVertex3f(                                DISTANCE_SCANNER_RADIUS,  DISTANCE_SCANNER_SENSOR_HALF_WIDTH, DISTANCE_SCANNER_ELEVATION + DISTANCE_SCANNER_HEIGHT);
00807       glVertex3f(                                DISTANCE_SCANNER_RADIUS,  DISTANCE_SCANNER_SENSOR_HALF_WIDTH, DISTANCE_SCANNER_ELEVATION);
00808       /* South face */
00809       glVertex3f(                                DISTANCE_SCANNER_RADIUS, -DISTANCE_SCANNER_SENSOR_HALF_WIDTH, DISTANCE_SCANNER_ELEVATION + DISTANCE_SCANNER_HEIGHT);
00810       glVertex3f(                                DISTANCE_SCANNER_RADIUS, -DISTANCE_SCANNER_SENSOR_HALF_WIDTH, DISTANCE_SCANNER_ELEVATION);
00811       /* East face */
00812       glNormal3f(1.0f, 0.0f, 0.0f);
00813       glVertex3f(DISTANCE_SCANNER_SENSOR_DEPTH + DISTANCE_SCANNER_RADIUS, -DISTANCE_SCANNER_SENSOR_HALF_WIDTH, DISTANCE_SCANNER_ELEVATION + DISTANCE_SCANNER_HEIGHT);
00814       glVertex3f(DISTANCE_SCANNER_SENSOR_DEPTH + DISTANCE_SCANNER_RADIUS, -DISTANCE_SCANNER_SENSOR_HALF_WIDTH, DISTANCE_SCANNER_ELEVATION);
00815       /* North face */
00816       glNormal3f(0.0f, 1.0f, 0.0f);
00817       glVertex3f(DISTANCE_SCANNER_SENSOR_DEPTH + DISTANCE_SCANNER_RADIUS,  DISTANCE_SCANNER_SENSOR_HALF_WIDTH, DISTANCE_SCANNER_ELEVATION + DISTANCE_SCANNER_HEIGHT);
00818       glVertex3f(DISTANCE_SCANNER_SENSOR_DEPTH + DISTANCE_SCANNER_RADIUS,  DISTANCE_SCANNER_SENSOR_HALF_WIDTH, DISTANCE_SCANNER_ELEVATION);
00819       /* West face */
00820       glNormal3f(-1.0f, 0.0f, 0.0f);
00821       glVertex3f(                                DISTANCE_SCANNER_RADIUS,  DISTANCE_SCANNER_SENSOR_HALF_WIDTH, DISTANCE_SCANNER_ELEVATION + DISTANCE_SCANNER_HEIGHT);
00822       glVertex3f(                                DISTANCE_SCANNER_RADIUS,  DISTANCE_SCANNER_SENSOR_HALF_WIDTH, DISTANCE_SCANNER_ELEVATION);
00823       glEnd();
00824    }
00825 
00826    /****************************************/
00827    /****************************************/
00828 
00829    void CQTOpenGLFootBot::RenderDistanceScanner() {
00830       /* Set material */
00831       SetWhitePlasticMaterial();
00832       /* Draw only side surface */
00833       CVector2 cVertex(DISTANCE_SCANNER_RADIUS, 0.0f);
00834       CRadians cAngle(CRadians::TWO_PI / m_unVertices);
00835       CVector2 cNormal(1.0f, 0.0f);
00836       glBegin(GL_QUAD_STRIP);
00837       for(GLuint i = 0; i <= m_unVertices; i++) {
00838          glNormal3f(cVertex.GetX(), cVertex.GetY(), 0.0f);
00839          glVertex3f(cVertex.GetX(), cVertex.GetY(), DISTANCE_SCANNER_ELEVATION + DISTANCE_SCANNER_HEIGHT);
00840          glVertex3f(cVertex.GetX(), cVertex.GetY(), DISTANCE_SCANNER_ELEVATION);
00841          cVertex.Rotate(cAngle);
00842          cNormal.Rotate(cAngle);
00843       }
00844       glEnd();
00845    }
00846 
00847    /****************************************/
00848    /****************************************/
00849 
00850    void CQTOpenGLFootBot::RenderIMX() {
00851       /* Set material */
00852       SetWhitePlasticMaterial();
00853       CVector2 cVertex(IMX_MODULE_RADIUS, 0.0f);
00854       CRadians cAngle(-CRadians::TWO_PI / m_unVertices);
00855       /* Bottom part */
00856       glBegin(GL_POLYGON);
00857       glNormal3f(0.0f, 0.0f, -1.0f);
00858       for(GLuint i = 0; i <= m_unVertices; i++) {
00859          glVertex3f(cVertex.GetX(), cVertex.GetY(), IMX_MODULE_ELEVATION);
00860          cVertex.Rotate(cAngle);
00861       }
00862       glEnd();
00863       /* Side surface */
00864       cAngle = -cAngle;
00865       CVector2 cNormal(1.0f, 0.0f);
00866       cVertex.Set(IMX_MODULE_RADIUS, 0.0f);
00867       glBegin(GL_QUAD_STRIP);
00868       for(GLuint i = 0; i <= m_unVertices; i++) {
00869          glNormal3f(cNormal.GetX(), cNormal.GetY(), 0.0f);
00870          glVertex3f(cVertex.GetX(), cVertex.GetY(), IMX_MODULE_ELEVATION + IMX_MODULE_HEIGHT);
00871          glVertex3f(cVertex.GetX(), cVertex.GetY(), IMX_MODULE_ELEVATION);
00872          cVertex.Rotate(cAngle);
00873          cNormal.Rotate(cAngle);
00874       }
00875       glEnd();
00876       /* Top part */
00877       /* Set material */
00878       SetCircuitBoardMaterial();
00879       glBegin(GL_POLYGON);
00880       glNormal3f(0.0f, 0.0f, 1.0f);
00881       cVertex.Set(IMX_MODULE_RADIUS, 0.0f);
00882       for(GLuint i = 0; i <= m_unVertices; i++) {
00883          glVertex3f(cVertex.GetX(), cVertex.GetY(), IMX_MODULE_ELEVATION + IMX_MODULE_HEIGHT);
00884          cVertex.Rotate(cAngle);
00885       }
00886       glEnd();
00887    }
00888 
00889    /****************************************/
00890    /****************************************/
00891 
00892    void CQTOpenGLFootBot::RenderBeacon() {
00893       CVector2 cVertex(BEACON_RADIUS, 0.0f);
00894       CRadians cAngle(-CRadians::TWO_PI / m_unVertices);
00895       /* Bottom part */
00896       glBegin(GL_POLYGON);
00897       glNormal3f(0.0f, 0.0f, -1.0f);
00898       for(GLuint i = 0; i <= m_unVertices; i++) {
00899          glVertex3f(cVertex.GetX(), cVertex.GetY(), BEACON_ELEVATION);
00900          cVertex.Rotate(cAngle);
00901       }
00902       glEnd();
00903       /* Side surface */
00904       cAngle = -cAngle;
00905       CVector2 cNormal(1.0f, 0.0f);
00906       cVertex.Set(BEACON_RADIUS, 0.0f);
00907       glBegin(GL_QUAD_STRIP);
00908       for(GLuint i = 0; i <= m_unVertices; i++) {
00909          glNormal3f(cNormal.GetX(), cNormal.GetY(), 0.0f);
00910          glVertex3f(cVertex.GetX(), cVertex.GetY(), BEACON_ELEVATION + BEACON_HEIGHT);
00911          glVertex3f(cVertex.GetX(), cVertex.GetY(), BEACON_ELEVATION);
00912          cVertex.Rotate(cAngle);
00913          cNormal.Rotate(cAngle);
00914       }
00915       glEnd();
00916       /* Top part */
00917       glBegin(GL_POLYGON);
00918       glNormal3f(0.0f, 0.0f, 1.0f);
00919       cVertex.Set(BEACON_RADIUS, 0.0f);
00920       for(GLuint i = 0; i <= m_unVertices; i++) {
00921          glVertex3f(cVertex.GetX(), cVertex.GetY(), BEACON_ELEVATION + BEACON_HEIGHT);
00922          cVertex.Rotate(cAngle);
00923       }
00924       glEnd();
00925    }
00926 
00927    /****************************************/
00928    /****************************************/
00929 
00930    void CQTOpenGLFootBot::RenderCamera() {
00931       /* Set material */
00932       SetWhitePlasticMaterial();
00933       CVector2 cVertex(CAMERA_RADIUS, 0.0f);
00934       CRadians cAngle(-CRadians::TWO_PI / m_unVertices);
00935       /* Bottom part */
00936       glBegin(GL_POLYGON);
00937       glNormal3f(0.0f, 0.0f, -1.0f);
00938       for(GLuint i = 0; i <= m_unVertices; i++) {
00939          glVertex3f(cVertex.GetX(), cVertex.GetY(), CAMERA_ELEVATION);
00940          cVertex.Rotate(cAngle);
00941       }
00942       glEnd();
00943       /* Side surface */
00944       cAngle = -cAngle;
00945       CVector2 cNormal(1.0f, 0.0f);
00946       cVertex.Set(CAMERA_RADIUS, 0.0f);
00947       glBegin(GL_QUAD_STRIP);
00948       for(GLuint i = 0; i <= m_unVertices; i++) {
00949          glNormal3f(cNormal.GetX(), cNormal.GetY(), 0.0f);
00950          glVertex3f(cVertex.GetX(), cVertex.GetY(), CAMERA_ELEVATION + CAMERA_HEIGHT);
00951          glVertex3f(cVertex.GetX(), cVertex.GetY(), CAMERA_ELEVATION);
00952          cVertex.Rotate(cAngle);
00953          cNormal.Rotate(cAngle);
00954       }
00955       glEnd();
00956       /* Top part */
00957       glBegin(GL_POLYGON);
00958       glNormal3f(0.0f, 0.0f, 1.0f);
00959       cVertex.Set(CAMERA_RADIUS, 0.0f);
00960       for(GLuint i = 0; i <= m_unVertices; i++) {
00961          glVertex3f(cVertex.GetX(), cVertex.GetY(), CAMERA_ELEVATION + CAMERA_HEIGHT);
00962          cVertex.Rotate(cAngle);
00963       }
00964       glEnd();
00965    }
00966 
00967    /****************************************/
00968    /****************************************/
00969 
00970    class CQTOpenGLOperationDrawFootBotNormal : public CQTOpenGLOperationDrawNormal {
00971    public:
00972       void ApplyTo(CQTOpenGLWidget& c_visualization,
00973                    CFootBotEntity& c_entity) {
00974          static CQTOpenGLFootBot m_cModel;
00975          c_visualization.DrawRays(c_entity.GetControllableEntity());
00976          c_visualization.DrawEntity(c_entity.GetEmbodiedEntity());
00977          m_cModel.Draw(c_entity);
00978       }
00979    };
00980 
00981    class CQTOpenGLOperationDrawFootBotSelected : public CQTOpenGLOperationDrawSelected {
00982    public:
00983       void ApplyTo(CQTOpenGLWidget& c_visualization,
00984                    CFootBotEntity& c_entity) {
00985          c_visualization.DrawBoundingBox(c_entity.GetEmbodiedEntity());
00986       }
00987    };
00988 
00989    REGISTER_QTOPENGL_ENTITY_OPERATION(CQTOpenGLOperationDrawNormal, CQTOpenGLOperationDrawFootBotNormal, CFootBotEntity);
00990 
00991    REGISTER_QTOPENGL_ENTITY_OPERATION(CQTOpenGLOperationDrawSelected, CQTOpenGLOperationDrawFootBotSelected, CFootBotEntity);
00992 
00993    /****************************************/
00994    /****************************************/
00995 
00996 }
```

---

Generated on 10 Jul 2018 for ARGoS by 
 1.6.1
